# Instrumented intervertebral or posterolateral fusion in elderly patients Clinical results of a single center

**DOI:** 10.1186/1471-2474-12-189

**Published:** 2011-08-18

**Authors:** Stefan Endres, Rene Aigner, Axel Wilke

**Affiliations:** 1 Department of orthopaedic surgery, Elisabeth-Klinik Bigge/Olsberg, Heinrich Sommer Strasse 4, 59939 Olsberg, Germany

**Keywords:** Posterolateral fusion, intervertebral fusion, elderly, outcome

## Abstract

**Background:**

Data on the clinical outcome after spinal fusion in the elderly patient are rare. To our knowledge there has been no clinical outcome assessment for instrumented spinal fusion in elderly patients comparing posterolateral fusion with intervertebral fusion. Aim of the current study was to evaluate the clinical outcome of elderly patients who underwent a spinal fusion procedure for degenerative spinal stenosis with instability. Main hypothesis was to test whether it is necessary to force an intervertebral fusion for a better clinical outcome in spinal fusion surgery of the elderly or not.

**Methods:**

Two subgroups - posterolateral fusion versus intervertebral fusion (cage vs. non-cage) were compared with regard to functional outcome, fusion rates and complications after a mean follow up of 3.8 years. Questionnaires were completed by the patients before surgery and at final follow-up. Changes in mean VAS and ODI scores (decrease from the baseline VAS and ODI scores) were compared.

**Results:**

The mean final follow up for all subjects was 3.8 years. Of the 114 patients, 2 patients were deceased at the time of the follow-up, 5 patients didn't want to participate and 107 patients completed the questionnaires. This resulted in an overall follow-up rate of 93%. At final follow-up, the patients demonstrated significant improvement in the VAS and ODI- compared with the preoperative scores in both groups. But overall there were no significant differences between both groups regarding the outcome assessment using the ODI and VAS.

**Conclusions:**

The results of this study shows that elderly patients aged over 75 benefit from instrumented lumbar fusion. The study suggests that there is no need to force an intervertebral fusion because elderly patients do not seem to benefit from this procedure.

## Background

As the population ages, the number of spinal fusions performed in elderly patients is continuously increasing. There is a historic conflict, however, concerning the safety and efficacy of spinal surgery in the elderly [1-3].

Instrumented spinal fusion in elderly patients has been problematized due to the risk of screw loosening and comorbidity. But data on clinical outcomes after spinal fusion in the elderly are rare. Limitations of most studies include small study populations, evaluation of perioperative complication rate, and radiographic assessment.

To our knowledge, previous studies have not assessed clinical outcomes for instrumented spinal fusion in elderly patients comparing posterolateral fusion with intervertebral fusion (Cage versus non-cage).

Therefore, the aim of the current study was to evaluate the clinical outcomes of elderly patients who underwent a spinal fusion procedure for degenerative spinal stenosis with degenerative scoliosis. Two subgroups - posterolateral fusion versus intervertebral fusion (cage vs. non-cage) were compared with regard to functional outcome, fusion rate, and complications after a mean follow-up of 3.8 years. Main hypothesis was to test wether it is necessary to force an intervertebral fusion for a better clinical outcome in spinal fusion surgery of the elderly or not.

## Methods

### Study Design

Charts and records of 114 patients who underwent spinal fusion surgery between January 2005 and June 2008 were reviewed. Inclusion criteria were age over 65, degenerative spinal stenosis with instability, presence of a completed questionnaire and surgery done by the author (S.E.). Follow up was performed by the author (S.E.) and co-author (R.A.).

**Figure 1:**
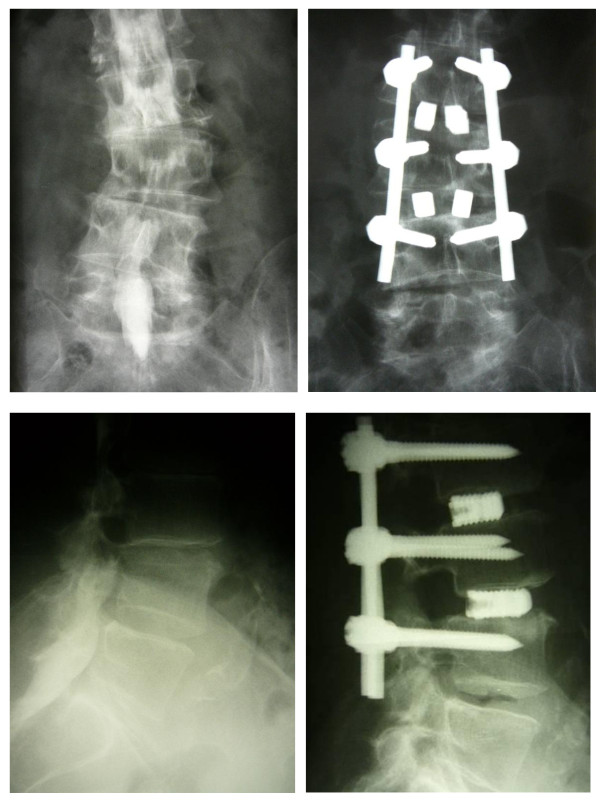
**Exemplary pre- and postoperative X-Ray of a degenerative spinal stenosis and scoliosis**. 75 years old female patient with degenerative spinal stenosis L2 to L4 and instability. Walking distance of 30 m.

All patients underwent fusion with pedicle screws and rod instrumentation (Tango RS, Fa. Ulrich, Germany) without or with intervertebral cages (PLIF: porous titanium cage, Fa. Aesculap, Germany) introduced by posterior lumbar interbody fusion. The bone graft for posterolateral fusion according to Wiltse was a mix of Endobone^® ^and autologous bone obtained from the decompression procedure. Laminectomy, partial resection of the facet and a foraminotomy was performed in all patients because of severe central (contrast-stop in functional myelography) and foraminal stenosis. The decision for the number of levels to fuse was based on functional myelography (degree of spinal stenosis and instability) as shown in Figure 1.

Postoperative management included early mobilization with bracing for 12 weeks on the first postoperative day.

The patient population was divided into 2 subgroups according to the type of fusion they underwent. Group 1 received instrumented posterior lumbar interbody fusion (porous titanium cage without bone grafts). This group included 54 patients [male/female: 36/18, mean age: 75.6 years (range, 66-78 years)]. Group 2 received a posterolateral lumbar fusion and included 60 patients [male/female: 38/22, mean age: 80.5 years (range, 73-88 years)]. The average number of fused levels was 1.5 ± 0.6 in group 1 (Range 1-4) and 1.8 ± 0.9 in group 2 (Range 1-5).

The review of anesthesia records for the patients showed that 35 patients in group 1 were of ASA (American Society of Anesthesiologists) class II and that 19 were of ASA class III. Nine (9) patients of group 2 were of ASA class I, 31 of ASA class II and 20 were of ASA class III.

See Table 1.

**Table 1:** Detailed group information and postoperative results

	instrumented PLIF	instrumented PLF
**Number of patients**	54	60
**Sex (male/female)**	36/18	38/22
**Age**	75.6 (66-78)	80.5 (73 - 88)
**Operation time**	143 min	88 min
**Blood loss**	760 ml (510 - 1100 ml)	470 ml (320 - 580 ml)
**Transfusion red cell units**	1.88 (0 - 5)	0.35 (0 - 2)
**ASA classification**		
Class I	0	9
Class II	35	31
Class III	19	20
**Number of levels fused**	1.5 (1 - 4)	1.8 (1 - 5)
**Degenerative spinal stenosis**		
monosegmental	39	37
2 segments	6	7
3 segments	7	10
4 segments	2	3
5 segments	0	3
**Length of hospital stay**	12.6	13.1
**Complications**		
Screw misplacement	1	0
Repeated decompression	2	1
Adjacent level disease	1	1
Pseudarthrosis	0	5
**Fusion rate**	41/54	46/60

### Outcome parameters

All patients who were still alive in July 2010 were called for assessment for the study by the postgraduate (R.A.). They were given the questionnaires including the Oswestry Disability Index (ODI) and a visual analogue scale (VAS) score to assess their functional outcome and quality of life at final follow up. Changes in mean VAS and ODI scores (decrease from the baseline VAS and ODI scores) were compared.

Fusion was assessed at final follow up on plain anteroposterior and lateral radiographs using the criteria suggested by Christensen et al. [4]

In additional, surgical time, need for red cell transfusions, and need for re-operation were documented.

### Ethical board statement

Ethical board approval of the University of Münster, Germany for the current study was given by the ethical board. [AZ 2010-218-f-s].

### Statistical Analysis

For an effect size of 0.5 in functional outcome with α = 0.05 and β = 0.80, calculations revealed that 53 patients would be needed in each group. The changes in the ODI and VAS were evaluated with use of the Wilcoxon-Mann-Whitney test. Chi quadrat testing was done for differentiation in fusion rate and complication.

## Results

The average intraoperative blood loss was recorded as a mean of 760 mL in group 1 (Range: 510 - 1100 mL) and 470 mL in group 2 (Range: 320 - 580 mL). Average time of surgery was 2 h 23 min for group 1 and 1 h 28 min for group 2. The need for transfusions (red cell units) in group 1 was on average 1.88 (Range: 0-5) and in group 2 on average 0.35 (Range: 0-2).

The mean final follow up for all subjects was 3.8 years. Of the 114 patients, 2 patients were deceased at the time of the follow-up, 5 patients didn't want to participate and 107 patients completed the questionnaires. This resulted in an overall follow-up rate of 93%.

The mean hospital stay was 12.6 days in Group 1 and 13.1 days in Group 2. The complication rate was almost the same in both groups. Revision surgeries were needed in group 1 with 2 repeat decompressions, 1 screw misplacement and 1 adjacent level disease. In group 2 there was a revision surgery needed in 5 cases due to pseudarthrosis mainly of L5/S1, 1 repeat decompression and 1 adjacent level disease.

At final follow-up, the patients in both groups demonstrated significant improvements in VAS and ODI-scores as compared to preoperative scores.

Group 1: The mean VAS score was 8.4 before surgery, and 5.0 at final follow up, showing 42% improvement. The mean ODI score was 53.05 ± 11.47 before surgery, and 38.59 ± 14.5., showing 29% improvement.

Group 2: The mean VAS score was 8.8 before surgery, and 5.2 at final follow up, showing 45% improvement. The mean ODI score was 53.43 ± 10.12 before surgery, and 39.01 ± 14.02, showing 27% improvement.

Overall, there were no significant differences between the two groups regarding outcome assessment using ODI and VAS. A sub-group analysis of patients who had complications wasn't done.

Fusion rate was determined by plain radiographs evaluated by the author (S.E.) and an independent radiologist. The fusion rate was similar in both groups with 41 out of 54 patients in group 1 showing successful fusion and 46 of the 60 subjects in group 2 showing successful fusion.

Chi-quadrat testing reveals no statistically significant correlation (at a significance level of 5%) between fusion rate and complication rate comparing the different surgical techniques (intervertebral fusion: X^2 ^= 0,0086; posterolateral fusion: X^2 ^= 0,5914).

See Figure 2 and 3.

**Figure 2:**
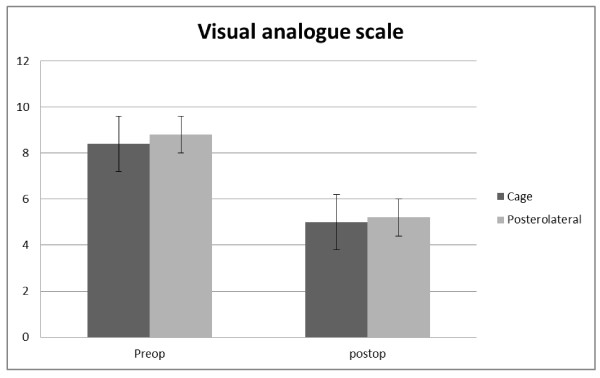
**Course of VAS**.

**Figure 3:**
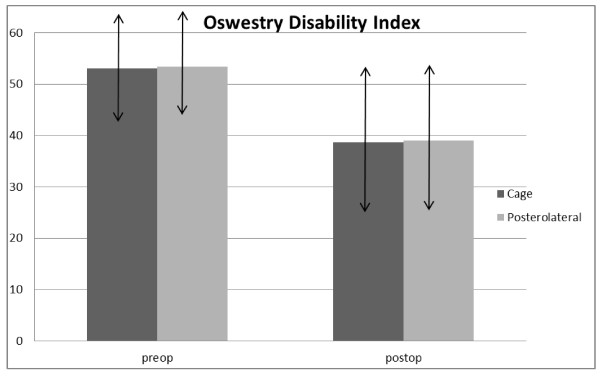
**Course of ODI**.

## Discussion

The life expectancy of the general population continues to increase and is expected to reach 86.6 for women and 81.1 for men by 2050. The very elderly (75 years and older) population is about 4% and is predicted to rise by 2050 to 12-13% [5].

In the past, and sometimes even today, advanced age was considered a contraindication for elective spinal surgery, so older patients were treated conservatively. However, conservative treatment can lead to increasing physical pain and limitations in quality of life [6,7].

The surgical procedures vary from laminotomy or wide central laminectomy alone to an anterior release with posterior decompression and fusion with instrumentation. Because of advanced age, medical co-morbidities lower bone density, and more spinal stiffness the surgical risks and complications are higher. The main objective of the surgical selection is to achieve the greatest benefit with the least complex intervention.

Reviewing the literature, there are many studies dealing with the clinical outcome after spinal fusion surgery of the elderly [8-13].

But the literature reveals different opinions about fusion surgery in the elderly, especially how to treat degenerative spinal stenosis with concomitant instability. The question of whether it is necessary to do an intervertebral fusion by implanting a cage or a posterolateral fusion (deposition of bone, bone substitutes) is sufficient remains controversial.

The literature shows that complication rates after surgery of the lumbar spine in elderly patients vary from 8 to 80%, with further variation in the rate of minor complications that do not lead to prolongation of hospital stay which usually account for more than half of the complications [2,8-10,14-18].

However, data on the clinical outcome after spinal fusion in the elderly patient are rare. Existing studies have either small numbers of patients, are focused on the perioperative complication rate [1,15,17], or put their emphasis on the radiologic outcome.

Therefore the aim of the current study was to evaluate clinical outcomes of patients over 65 who underwent a spinal fusion procedure for degenerative spinal stenosis with concomitant instability. Two subgroups - posterolateral fusion versus intervertebral fusion (cage vs. non-cage) were compared with regard to functional outcome, fusion rate and major complications after a mean follow up of 3.8 years.

Looking at the most recent literature the existing studies show that the assessment of success after spinal fusion surgery in the geriatric patient has been focused on the perioperative complication rate, with little attention directed toward improvement in function, quality of life, patient satisfaction, or improvement in perceptions of pain and the need for medication.

Okuda et al were the first to assess a large number of elderly patients on the basis of health status questionnaires after posterior lumbar interbody fusion, but they compared patients with a mean age of 74 to patients with a mean age of 59. They found no differences in the clinical and functional outcomes between the two groups and stated that posterior lumbar interbody fusion is a safe and accurate procedure for geriatric patients [19].

Andersen et al reported that superior outcomes after lumbar spinal fusion in elderly patients can be achieved using instrumentation, but the aim of their study was to compare instrumented and non-instrumented lumbar spinal fusions performed using fresh frozen allograft in patients older than 60 years with regard to functional outcome and fusion rates. The outcome was better for patients in which a solid fusion was obtained. However, instrumentation was associated with a larger number of additional surgeries, which resulted in a lesser degree of improvement [11].

A study by Glassmann et al support the efficacy of lumbar decompression and fusion in selected patients over 65 years of age. In their retrospective evaluation they had an improvement in SF-36 and ODI at a 2 year follow up which is in line with our study results [12].

Another study by Becker et al investigated the clinical outcome in elderly patients who underwent spinal fusion with pedicle screws and rod instrumentation with or without intervertebral cages introduced by posterior lumbar interbody fusion. They were able to demonstrate that e elderly patients benefit from spinal fusion but they do not distinguish between the two methods [13].

To our knowledge, there is no other study that compares the clinical and functional outcomes after instrumented spinal surgery in the elderly with regard to intervertebral fusion or posterolateral fusion.

Using the ODI and VAS, we found no differences between the two subgroups (cage versus posterolateral fusion) of elderly patients. Overall a significant improvement in life quality was found at final follow up regardless the used fusion technique or whether a revision procedure was done in the meantime. A subgroup analysis of the patients who were in need of a revision surgery was not done. But looking at the ODI and VAS they don't have obvious lower scores.

With regard to intraoperative blood loss, need for transfusion of red cell units and surgical time, there was a significant difference between the two subgroups that showed superiority for the instrumented posterolateral fusion group.

The present study has some limitations. The duration of the follow-up was relatively short. The long-term results of these surgical procedures are needed. The second issue is that there is little information available regarding fusion rates in elderly patients. The fusion rate in the present study was evaluated indirectly as conventional x-ray was used for follow-up. Owing to ethical issues concerning high radiation, CT scans were not performed. But whether osseous fusion rate correlates with the clinical outcome remains a controversial issue. It was demonstrated by Pfeiffer et al. that there is only a weak correlation between intervertebral fusion and the clinical outcome [20]. Additional no reported diagnostic technique, including fine-cut CT scans, has shown a high level of accuracy in predicting spinal fusion [21]. In contrast to a study by Inamdar et al [22] who recommended PLF over PLIF because of the simplicity of the procedure, lower complication rate and good clinical and radiological outcomes with a reported fusion rate of 100% in both groups we observed a higher rate of pseudarthrosis in the PLF group which made a revision surgery necessary and may negatively affect the clinical outcome.

The third issue is the sagittal balance. Even if several investigators have stressed the importance of maintaining sagittal balance to avoid lumbar "flat back," accelerated adjacent segment degeneration, pain, and inferior functional outcome only limited evidence exists on how sagittal alignment affects clinical outcome [23,24]. But even the use of intervertebral fusion devices with possibly improved restoration of sagittal spinal balance will not have an effect on clinical outcome as shown by the present study.

Explanation for this phenomenon is that in addition to sagittal balance, clinical outcomes of instrumented lumbar fusion in patients with degenerative lumbar spine disease are influenced by a variety of pathophysiologic factors, including residual compression of the neural tissues, recurrence of spinal canal stenosis, irreversible changes to the nerve root, or cauda equina.

## Conclusions

The results of this study show that elderly patients over 75 benefit from instrumented lumbar fusion. The study further suggests that there is no need to advocate for an intervertebral fusion because elderly patients do not seem to benefit from this procedure. With regard to clinical and functional outcomes, an instrumented posterolateral fusion is sufficient. Additionally, the intraoperative blood loss, need for transfusions and surgical time are clearly reduced for elderly patients receiving posterolateral fusion.

## Competing interests

There was no involvement of a manufacturer in this study.

### Financial competing interests

- In the past five years have you received reimbursements, fees, funding, or salary from an organization that may in any way gain or lose financially from the publication of this manuscript, either now or in the future? Is such an organization financing this manuscript (including the article-processing charge)? If so, please specify.

No.

- Do you hold any stocks or shares in an organization that may in any way gain or lose financially from the publication of this manuscript, either now or in the future? If so, please specify.

No.

- Do you hold or are you currently applying for any patents relating to the content of the manuscript? Have you received reimbursements, fees, funding, or salary from an organization that holds or has applied for patents relating to the content of the manuscript? If so, please specify.

No.

- Do you have any other financial competing interests? If so, please specify.

No.

### Non-financial competing interests

- Are there any non-financial competing interests (political, personal, religious, ideological, academic, intellectual, commercial or any other) to declare in relation to this manuscript? If so, please specify.

No.

## Authors' contributions

SE carried out the surgery, follow up and drafted the manuscript. RA has done the assessment of the patients. AW helped revising the manuscript and with statistics. All authors read and approved the final manuscript.

## Pre-publication history

The pre-publication history for this paper can be accessed here:

http://www.biomedcentral.com/1471-2474/12/189/prepub
